# Antinociceptive Effects of Shenling Baizhu through PI3K-Akt-mTOR Signaling Pathway in a Mouse Model of Bone Metastasis with Small-Cell Lung Cancer

**DOI:** 10.1155/2020/4121483

**Published:** 2020-06-22

**Authors:** Zhe Feng, Ziyi Feng, Jie Han, Weimin Cheng, Bo Su, Jian Mo, XinJian Feng, Sitan Feng, GuoJian Chen, Peng Huang, Long Huang, Zhenwei Cui

**Affiliations:** ^1^Department of Sports and Joint Surgery, Ruikang Hospital Affiliated to Guangxi University of Chinese Medicine, Nanning 530011, China; ^2^Guangxi University of Chinese Medicine, Nanning, China; ^3^Department of Blood Specialty, The First Affiliated Hospital of Guangxi University of Chinese Medicine, Nanning 530023, China

## Abstract

Shenling Baizhu additive powder (SLBZ-AP), a formulation of a variety of natural medicinal plants, has clinical efficacy in treating cancers in previous studies. We explored the effect of SLBZ-AP in bone metastasis of lung cancer (BMLC) mice, and the possible mechanism involved was further investigated in the present study. Mice model of BMLC was made and treated with SLBZ-AP. Pain behavioral tests were performed to explore the effect on BMLC-induced pain in mice. TUNEL staining was used to investigate apoptosis. The mRNA expression of markers in the PI3K/Akt/mTOR pathway was measured by qPCR, and protein expression was detected by western blotting and immunohistochemistry analysis. SLBZ-AP relieved BMLC-induced pain and prolonged animals' survival, promoted cell apoptosis in the marrow from the tibia of BMLC mice, and inhibited mRNA and protein expression of AKT, mTOR, P70S6, and VEGF, as well as protein expression of p-AKT, p-mTOR, p-P70S6, and VEGF upregulation in the marrow of tibia induced by BMLC, an effect which was similar to rapamycin. Our results suggested that SLBZ-AP may have antinociceptive effect and prolong survival of BMLC mice at least partially by inhibiting cell proliferation and promoting apoptosis through the PI3K/Akt/mTOR signaling pathway. SLBZ-AP may be a potential candidate for BMLC therapy.

## 1. Introduction

Small- cell lung cancer (SCLC) is one form of lung cancer, which is the leading cause of cancer-related deaths worldwide [1]. Lung cancer tends to develop into bone metastases (BMLC) strongly and about 30–40% of lung cancer patients develop bone metastasis [2–4]. Development of BMLC is associated with poor prognosis and high morbidity and BMLC-induced pain has strong impact on the patients' quality of life [5, 6]. Therefore, prevention of BMLC and relieving the pain to improve lung cancer patients' quality of life are critical. It is necessary to find safe and effective drug therapies for BMLC currently. PI3K/Akt/mTOR signaling pathway plays a pivotal role in a variety of biological activities to regulate cell proliferation, survival, and migration [7, 8]. The PI3K/AKT/mTOR axis commonly contributes to possible mechanisms of oncogenic transformation including stimulation of proliferation, survival, invasion/metastasis, and metabolic reprogramming, as well as suppression of autophagy [9]. PI3K/Akt/mTOR signaling pathway is one of the major signaling cascades which is frequently activated in various human cancers including lung cancer [10, 11]. It has been confirmed by researchers that suppression of PI3K/Akt/mTOR signaling pathway activation may inhibit cells proliferation and metastasis in lung cancer [12–15]. PI3K/Akt/mTOR signaling pathway has been considered a promising therapeutic target.

Shenling Baizhu Powder (SLBZ-AP) is a well-known Chinese medicine formula. The prescription principle of SLBZ-AP is to replenish Qi and invigorate the spleen [16], resolve dampness [17], and relieve diarrhea [18] according to traditional Chinese medicine (TCM) theory [19]. SLBZ-AP is reported to be commonly used to treat patients with shortness of breath, poor appetite, abdominal distension, loose stool, prolapsed anus, lassitude, dysphasia, and spontaneous sweating [20, 21]. It was reported that SLBZ-AP could increase abundance of beneficial gut microbiota and decrease levels of LPS in the portal vein of rats to indicate effects on nonalcoholic fatty liver disease (NAFLD) [22]. SLBZ-AP induced TNF-*α*, IL-1, and IL-6 significantly decreased in Kupffer cells of nonalcoholic steatohepatitis rats [23]. Studies showed that SLBZ-AP is also effective in curing chronic enteritis [24, 25]. Researchers reported that SLBZ-AP plays an important role in tumor therapy. SLBZ-AP could improve spleen deficiency syndrome after gastric cancer [26]. SLBZ-AP treatment reduced the death rate of mice and decreased the incidence and multiplicity of colonic neoplasms, as well as lowered myeloid-derived suppressor cells infiltration and alleviated TGF-*β*1 induced epithelial mesenchymal transition (EMT) to exert its effects of anticolitis associated colorectal cancer [19]. SLBZ-AP combined with chemotherapy in postoperative treatment can improve the patient's efficacy after colon cancer surgery while not showing more side effects [27]. SLBZ-AP inhibit tumor growth and increase IL-2, IFN-*γ* and TNF-*α* in mice with xenografted hepatocellular cancers [28]. SLBZ-AP combined with gefitinib/erlotinib in the treatment of advanced lung cancer showed more efficacy in clinical practice [29]. According to some researchers, it has been found that SLBZ-AP treatment had a good curative effect on lung cancer and improved quality of life of lung cancer patients [29, 30]. BMLC is associated with poor prognosis and high morbidity and patients' quality of life was strongly affected by BMLC-induced pain. In our previous study, SLBZ-AP tends to show effects on BMLC patients [31, 32].

In the present study, we investigated the effect of SLBZ-AP on BMLC-induced pain and survival in BMLC model mice and further explored the possible signaling pathway involved, as PI3K-Akt-mTOR. The results of the present study provided prospective TCM therapeutic approaches for BMLC and improving BMLC-induced pain.

## 2. Materials and Methods

### 2.1. Cell Lines and Culture

Human small-cell lung cancer (SCLC) cell line SBC-5 was obtained from Japanese Collection of Research Bioresources Cell Bank (JCRB0819, Japan) and was maintained in RPMI-1640 medium (Gibco, Thermo Fisher Scientific, Inc., Waltham, MA, USA) supplemented with 10% fetal bovine serum (FBS) (Gibco, Thermo Fisher Scientific, Inc., Waltham, MA, USA). Cells were incubated at 37°C in an atmosphere with 5% CO_2_.

### 2.2. Drugs

Shenling Baizhu additive powder (SLBZ-AP) contains twenty-eight species of traditional Chinese medicines (TCM), named according to Chinese Pharmacopoeia [33], as listed in Table 1 (supplied by Pharmacy of Ruikang Hospital Affiliated to Guangxi University of Chinese Medicine, China). All the TCM involved in the formula are crushed into fine powder, sieved, and mixed to obtain SLBZ-AP. According to a previous study [22], SLBZ-AP was dissolved in distilled water to prepare decoction and stored at −4°C. The water decoction was characterized according to the method mentioned in the previous reports [34, 35]. All the plant materials included in Shenling Baizhu used in our study are identified in laboratory department of Pharmacy of Ruikang Hospital Affiliated to Guangxi University of Chinese Medicine according to Chinese Pharmacopoeia. All the voucher specimens of the material have been deposited in Guangxi University of Chinese Medicine.

### 2.3. Animals and Experimental Design

The animal studies were conducted and approved by Medical Animal Ethics Committee of Guangxi University of Chinese Medicine. A total of 30 male BALB/c nude mice (6–8 weeks old) were purchased from Shanghai Experimental Animal Center of Chinese Academy of Science (Shanghai, China). All the animals had free access to food and water in a controlled environment with temperature of 25 ± 2°C, 50 ± 10% humidity, and 12 h light/dark cycle. After adaptive feeding for 1 week, the mice were randomly divided into 6 groups with 5 mice per group: (1) sham group which was injected with sterile PBS (pH 7.2) into the right tibia of each mouse; (2) vehicle group, where the mice model of bone metastasis of lung cancer (BMLC) was made according to the previous reports [36]; briefly, 2 × 10^6^ SBC-5 cells/mouse were injected into the right tibia of the animal which then received 0.9% saline i.g. (volume was equivalent to therapeutic drugs); (3) low dose group (L-D), where BMLC model mice were treated with 15 g·kg^−1^·day^−1^ for 2  weeks; (4) medium dose group (M-D), where BMLC model mice were treated with 30 g·kg^−1^·day^−1^ for 2 weeks; (5) high dose group (H-D), where BMLC model mice were treated with 60 g·kg^−1^·day^−1^ for 2 weeks; and (6) rapamycin group, where BMLC model mice were treated with 4 mg·kg^−1^·day^−1^ for 2 weeks. The dosage was adjusted according to the mice weight measured daily. The survival of animals was observed daily. The mice were euthanized with 100% carbon dioxide and sacrificed at 12 h after the last treatment and the right tibia specimens were collected and decalcification was performed followed by paraffin embedding and histopathological examination.

### 2.4. Pain Behavioral Tests

Pain intensity to mechanical stimulus was measured using Von Frey monofilaments. Mice were placed on an elevated wire framework in a transparent box, and then different pressure intensities (equivalent to 1 to 30 g) of von Frey filaments were applied to the plantar surface of light hind. Each filament, from the smallest von Frey filament and increasing gradually, was applied to the plantar surface of the hind paw until the filament bent. The lowest pressure filament in two out of three applications that induced paw licking, paw lifting, and paw withdrawal was used to determine paw withdrawal mechanical threshold (g) (PWMT).

### 2.5. RNA Extraction and Quantitative Real-Time PCR Analysis (qPCR)

Total RNA was isolated from marrow of tibia specimens from mice using Trizol (TaKaRa, Japan) according to the manufacturer's instructions. Reverse transcription was performed using the reverse transcription kit (Invitrogen, USA). The same amounts of RNA were added to a reverse transcriptase reaction mix with oligo-dT as a primer. Quantitative real-time PCR analyses were performed using the SYBR Green PCR Master Mix kit (Thermo Scientific, USA) according to the manufacturer's instructions. After an initial incubation at 95°C for 2 min, amplification was performed for 40 cycles at 95°C for 15 s, 60°C for 20 s, and 72°C for 20 s. The corresponding specific primers were showed in Table 2.

### 2.6. Western Blot Analysis

The marrow of tibia specimens were lysed in cold RIPA buffer and centrifuged at 10,000 ×g for 10 min at 4°C. The lysates were then run on 10% SDS-PAGE gel and transferred onto PVDF membranes (Millipore, Shanghai, China).After blocking the membranes in Tris-buffered saline (TBS) buffer with 5% skim milk for 6 h at room temperature, the membranes were incubated with primary antibodies against *β*-actin (Santa, USA), p-AKT (CST, USA), p-mTOR (CST, USA), p-P70S6k (CST, USA), and VEGF (CST, USA) (1 : 500 to 1 : 1,000) overnight at 4°C.Then, the membranes were washed and incubated with horseradish peroxidase-conjugated secondary antibody for 1 h at room temperature. Immunoblots were detected using enhanced chemiluminescence reagents (Invitrogen, USA) and analyzed by Image J software to quantify. The results were normalized to *β*-actin to correct for loading.

### 2.7. Histological Analysis

The marrow of tibia specimens was harvested and then stored in 10% formal saline and embedded in paraffin blocks. 3–5 *μ*m sections were prepared from the paraffin blocks for further staining.

Sections of the marrow of tibia specimens were stained with hematoxylin and eosin (HE) in our research to visualize the tissue morphology.

For immunohistochemistry analysis, sections of the marrow of tibia specimens were deparafinized with xylene and then gradient alcohol was used to hydrate. The endogenous peroxidase activity was inhibited using hydrogen peroxide accompanied with a protein blocking agent. Next, the sections were incubated with the appropriate primary antibodies against p-AKT (CST, USA), p-mTOR (CST, USA), p-P70S6k (CST, USA), and VEGF (CST, USA) (1 : 500 to 1 : 1,000) overnight at 4°C and with the secondary antibody for 1hsubsequently. Nucleus was counterstained using hematoxylin. Photomicrograph of all stained sections photomicrograph was taken (Nikon, Japan).

### 2.8. TUNEL Staining

The marrow of tibia sections was permeabilized with 0.1% Triton ×100 (Beyotime, Shanghai, China). An in situ cell death detection kit (Roche, Mannheim, Germany) was used to perform TUNEL staining according to the manufacturer's instructions. And DAPI was used to counterstain the nucleus. Images were captured using the microscope.

### 2.9. Statistical Analysis

All data are presented as mean ± SD from the least three independent experiments. All data were analyzed using the GraphPad Prism 8 software (USA). Differences between two groups were analyzed using Student's *t*-test. Differences were considered statistically significant when *p* value <0.05. Survival rate was analyzed with Kaplan-Meier survival analysis.

## 3. Results

### 3.1. Shenling Baizhu Additive Powder (SLBZ-AP) Relieves Bone Metastasis of Lung Cancer- (BMLC-) Induced Pain and Prolongs Animals' Survival

**Figure 1:**
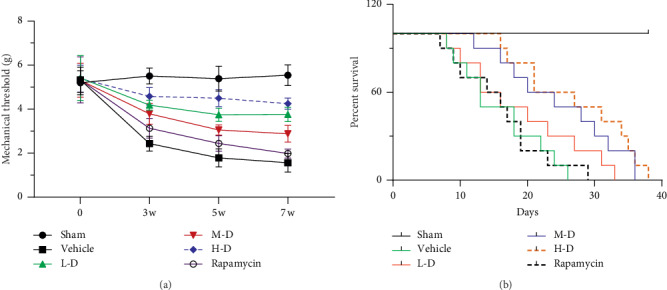
The effect of Shenling Baizhu Additive Powder (SLBZ-AP) on pain and survival of mice with bone metastasis of lung cancer (BMLC). (a) Pain behavior of mice was analyzed using paw withdrawal mechanical threshold (g) (PWMT). (b) The survival of animals was observed daily and percent survival was calculated. Vehicle: BMLC mice treated with placebo (saline). L-D: BMLC mice treated with 15 g·kg^−1^·day^−1^ SLBZ-AP for 2 weeks. M-D: BMLC mice treated with 30 g·kg^−1^·day^−1^ SLBZ-AP for 2 weeks. H-D: BMLC mice treated with 60 g·kg^−1^·day^−1^ SLBZ-AP for 2 weeks. Rapamycin: BMLC mice treated with 4 mg·kg^−1^·day^−1^ rapamycin for 2 weeks.

We investigated the effect of SLBZ-AP on pain and survival in BMLC mice. Paw withdrawal mechanical threshold (g) (PWMT) is used to analyze pain behavior. Compared with the sham group, pain sensitization was significantly decreased in mice of the placebo group (vehicle) (*p* < 0.01) (Figure 1(a)). 15 (L-D), 30 (M-D), 60 (H-D) g·kg^−1^·day^−1^ doses of SLBZ-AP showed relieved effect on BMLC induced pain, especially H-D treatment. It is remarkable that SLBZ-AP showed significant relieved effect on BMLC induced pain compared with rapamycin.

We further explored the effect of SLBZ-AP on survival of mice with BMLC. As shown in Figure 1(b), M-D (30 g·kg^−1^·day^−1^) and H-D (60 g·kg^−1^·day^−1^) treatment prolonged effect while L-D (15 g·kg^−1^·day^−1^) treatment has no change on survival of mice with BMLC. Rapamycin showed almost no effect on survival of mice with BMLC, which was similar to the result of pain behavioral analysis.

### 3.2. Effect of SLBZ-AP on Alleviating Bone Metastasis of Lung Cancer (BMLC) in Mice

**Figure 2:**
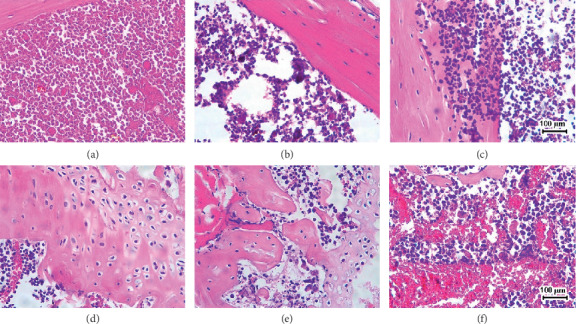
H&E staining of the marrow of tibia in mice with BMLC. Tumor cells in H&E staining images can be identified by deep purple color and irregular nuclear shape. Vehicle: BMLC mice treated with placebo (saline). L-D: BMLC mice treated with 15 g·kg^−1^·day^−1^ SLBZ-AP for 2 weeks. M-D: BMLC mice treated with 30 g·kg^−1^·day^−1^ SLBZ-AP for 2 weeks. H-D: BMLC mice treated with 60 g·kg^−1^·day^−1^ SLBZ-AP for 2 weeks. Rapamycin: BMLC mice treated with 4 mg·kg^−1^·day^−1^ rapamycin for 2 weeks. (a) Sham. (b) Vehicle. (c) L-D. (d) M-D. (e) H-D. (f) Rapamycin.

Tumor cells can be identified by deep purple color and irregular nuclear shape in images of H&E staining. It is shown in Figure 2 that more tumor cells were found in the marrow from tibia of BMLC mice compared with the sham group. Treatment with L-D, M-D, or H-D SLBZ-AP decreased tumor cells, and M-D and H-D showed effect more significantly. L-D SLBZ-AP and rapamycin have almost equivalent effects on BMLC.

### 3.3. SLBZ-AP Affects Apoptosis in BMLC Mice

**Figure 3:**
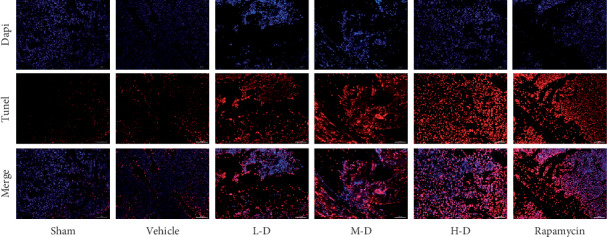
TUNEL staining of the marrow of tibia in mice with BMLC. Dapi was used to stain nucleus. TUNEL staining (red) indicates apoptosis. Vehicle: BMLC mice treated with placebo (saline). L-D: BMLC mice treated with 15 g·kg^−1^·day^−1^ SLBZ-AP for 2 weeks. M-D: BMLC mice treated with 30 g·kg^−1^·day^−1^ SLBZ-AP for 2 weeks. H-D: BMLC mice treated with 60 g·kg^−1^·day^−1^ SLBZ-AP for 2 weeks. Rapamycin: BMLC mice treated with 4 mg ·kg^−1^·day^−1^ rapamycin for 2 weeks.

TUNEL staining was performed to evaluate the effect of SLBZ-AP on apoptosis of the marrow from tibia of BMLC mice. Less apoptosis was observed in the sham or vehicle group. L-D, M-D, or H-D SLBZ-AP treatment promoted cells apoptosis in marrow from tibia of BMLC mice significantly (Figure 3). The effect of H-D SLBZ-AP was similar to that of rapamycin on cell apoptosis in the marrow from the tibia of BMLC mice.

### 3.4. Change in mRNA Expression of AKT, mTOR, P70S6, and VEGF after SLBZ-AP Treatment

**Figure 4:**
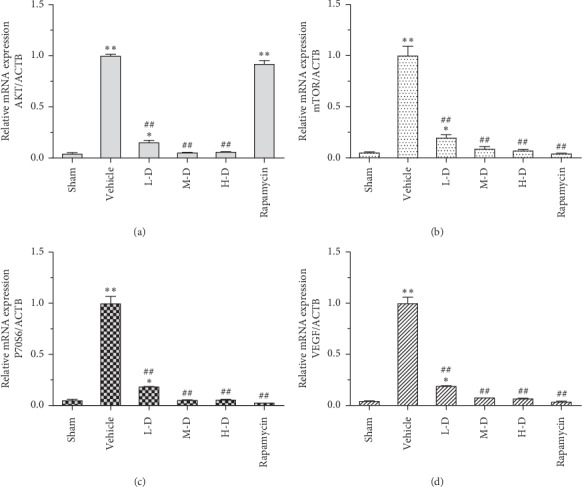
The mRNA expression of AKT, mTOR, P70S6, and VEGF in the marrow of tibia of mice with BMLC was measured by qPCR. (a) The AKT expression. (b) The mTOR expression. (c) The P70S6 expression. (d) The VEGF expression. Vehicle: BMLC mice treated with placebo (saline). L-D: BMLC mice treated with 15 g·kg^−1^·day^−1^ SLBZ-AP for 2 weeks. M-D: BMLC mice treated with 30 g·kg^−1^·day^−1^ SLBZ-AP for 2 weeks. H-D: BMLC mice treated with 60 g·kg^−1^·day^−1^ SLBZ-AP for 2 weeks. Rapamycin: BMLC mice treated with 4 mg·kg^−1^·day^−1^ rapamycin for 2 weeks.

qPCR assay was performed to detect the mRNA expression of AKT, mTOR, P70S6, and VEGF in the marrow of tibia from BMLC mice after SLBZ-AP treatment. BMLC induced the mRNA expression of AKT, mTOR, P70S6, and VEGF upregulation significantly (*p* < 0.01) (Figure 4). SLBZ-AP treatment downregulated AKT, mTOR, P70S6, or VEGF expression in the marrow of tibia from BMLC mice in a dose-dependent manner (15–60 g·kg^−1^·day^−1^) compared with the sham group. Rapamycin inhibited BMLC-induced increased expression of mTOR, P70S6, and VEGF particularly. There is no change of AKT expression in BMLC mice treated with rapamycin compared with the vehicle group (Figure 4(a)).

### 3.5. Change in Protein Expression of AKT, mTOR, P70S6, and VEGF after SLBZ-AP Treatment Analyzed by Immunohistochemistry

**Figure 5:**
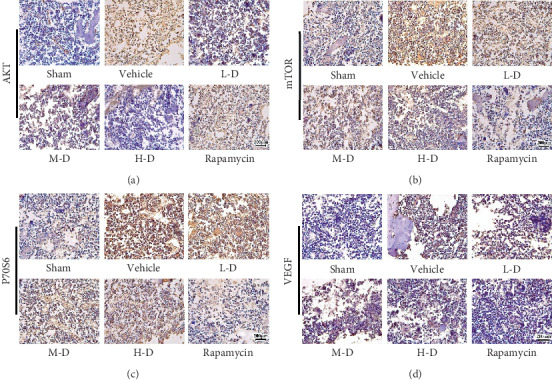
The protein expression of AKT, mTOR, P70S6, and VEGF in the marrow of tibia of mice with BMLC was analyzed by immunohistochemistry. (a) The AKT protein expression. (b) The mTOR protein expression. (c) The P70S6 protein expression. (d) The VEGF protein expression. Vehicle: BMLC mice treated with placebo (saline). L-D: BMLC mice treated with 15 g·kg^−1^·day^−1^ SLBZ-AP for 2 weeks. M-D: BMLC mice treated with 30 g·kg^−1^·day^−1^ SLBZ-AP for 2 weeks. H-D: BMLC mice treated with 60 g·kg^−1^·day^−1^ SLBZ-AP for 2 weeks. Rapamycin: BMLC mice treated with 4 mg·kg^−1^·day^−1^ rapamycin for 2 weeks.

The protein expression of AKT, mTOR, P70S6, and VEGF in the marrow of tibia in mice was analyzed using immunohistochemistry analysis. Compared with the sham group, BMLC induced AKT (Figure 5(a)), mTOR (Figure 5(b)), P70S6 (Figure 5(c)), and VEGF (Figure 5(d)) protein expression significantly. All the animals treated with SLBZ-AP (15–60 g·kg^−1^·day^−1^) showed decreasing protein expression of AKT, mTOR, P70S6, and VEGF. It is similar to the results of mRNA expression assay which showed that rapamycin inhibited mTOR, P70S6, and VEGF protein expression significantly and tended to show no effect on AKT compared with the vehicle group.

### 3.6. Change in Protein Expression of p-AKT, p-mTOR, p-P70S6, and VEGF after SLBZ-AP Treatment Detected by Western Blotting

**Figure 6:**
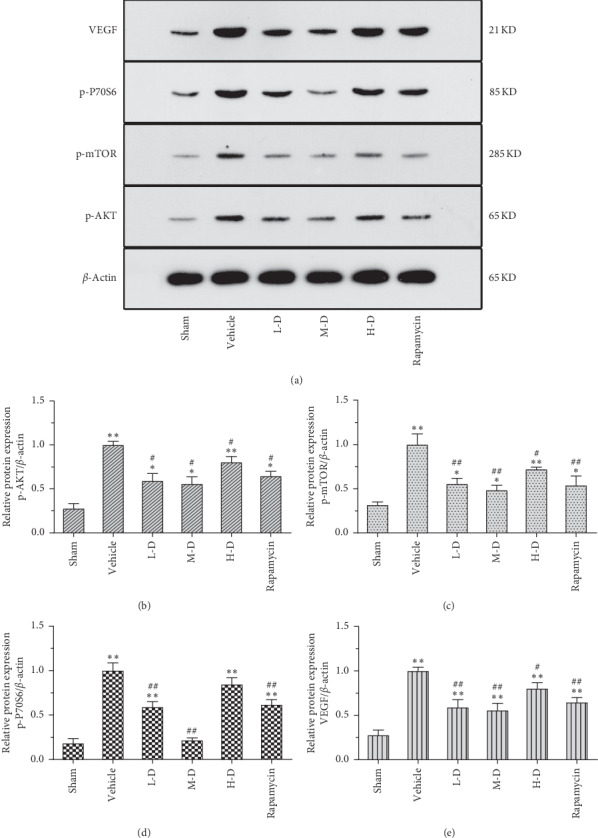
The protein expression of p-AKT, p-mTOR, p-P70S6, and VEGF in the marrow of tibia of mice with BMLC was analyzed by immunohistochemistry. (a) Western blots of p-AKT, p-mTOR, p-P70S6, and VEGF. (b) The p-AKT protein expression. (c) The p-mTOR protein expression. (d) The p-P70S6 protein expression. (e) The VEGF protein expression. Vehicle: BMLC mice treated with placebo (saline). L-D: BMLC mice treated with 15 g·kg^−1^·day^−1^ SLBZ-AP for 2 weeks. M-D: BMLC mice treated with 30 g·kg^−1^·day^−1^ SLBZ-AP for 2 weeks. H-D: BMLC mice treated with 60 g·kg^−1^·day^−1^ SLBZ-AP for 2 weeks. Rapamycin: BMLC mice treated with 4 mg·kg^−1^·day^−1^ rapamycin for 2 weeks.

We further measured the protein expression of p-AKT, p-mTOR, p-P70S6, and VEGF in the marrow of tibia in mice by western blotting. The protein expression of p-AKT, p-mTOR, p-P70S6, and VEGF was increased significantly in BMLC mice (*p* < 0.01) (Figure 6), which was downregulated by SLBZ-AP treatment, especially by 30 g·kg^−1^·day^−1^ SLBZ-AP (M-D). The effect of 60 g·kg^−1^·day^−1^ SLBZ-AP on p-AKT, p-mTOR, p-P70S6, and VEGF tends to be less than that of L-D and M-D SLBZ-AP. Rapamycin and L-D SLBZ-AP treatment indicated equivalent effect on p-AKT, p-mTOR, p-P70S6, and VEGF protein expression.

## 4. Discussion

Lung cancer is the leading cause of cancer-related deaths worldwide [1]. The incidence rate of lung cancer developing into bone metastases (BMLC) is higher, which is related to poor prognosis and high morbidity, and BMLC-induced pain affects the patients' quality of life strongly [5, 6]. Safe and effective drug therapies to prevent BMLC and relieve the pain to improve lung cancer patients' quality of life remain necessary currently. As a canonical and well-known Chinese medicine formula, SLBZ-AP is first described in “The Prescriptions of the Bureau of Taiping People's Welfare Pharmacy” in Song-dynasty [19] and contains twenty-eight species of TCM as Dangshen, Fuling, Baizhu, Gancao, and so on . There are chemical components as saccharides (Dang Shen, Fu Ling, Bai Bian Dou) [37, 38], glycosides (Shan Yao, Jie Geng, Shan Zhu Yu, Bai Shao) [39–42], and flavonoids (Bai Zhu, Gan Cao) [43, 44] mainly in SLBZ-AP. Saccharides have immunomodulation effects [37]. Glycosides as saponins [39] and flavonoids [44] have anti-inflammatory effects. It also has been reported that saponins could inhibit invasion and metastasis in colorectal cancer cell through NF-*κ*B signaling pathway and EMT [45]. Flavonoids also showed anticancer effect [46]. Dang gui could ameliorate skin inflammation [47] and has activities against cancer [48]. The other or the above mentioned drugs may have multiple as well as unclear chemical components which remain to be further explored. As reported in many previous studies, SLBZ-AP plays an important role in tumor therapy including lung cancer. SLBZ-AP combined with Gefitinib/Erlotinib had a good curative effect for advanced non-small-cell lung cancer with spleen deficiency [29]. SLBZ-AP treatment applied in the advanced non-small-cell lung cancer can improve the clinical efficacy, relieve clinical symptoms, and improve the quality of life of patients [30]. SLBZ-AP improve clinical chemotherapy benefit and quality of life of patients with non-small-cell lung cancer by upregulating the expression of VEGF and MMP-9. In addition, SLBZ-AP treatment has higher safety [49]. The effect of SLBZ-AP on BMLC-induced pain and survival of BMLC mice as well as involved signaling pathway was explored in our study.

Mice model of BMLC was made and treatment with SLBZ-AP was performed. Our data showed that SLBZ-AP relieves BMLC-induced pain and prolongs animals' survival. It is remarkable that SLBZ-AP showed significant relieved effect on BMLC-induced pain and survival of BMLC mice compared with rapamycin. H&E staining of the marrow of tibia in animals showed that M-D and H-D of SLBZ-AP has effect on inhibition of cell proliferation more significantly. Results of TUNEL staining indicated that L-D, M-D, or H-D of SLBZ-AP treatment promoted cells apoptosis in marrow from tibia of BMLC mice significantly. Our results suggested that SLBZ-AP have antinociceptive effect and may prolong survival of BMLC mice by inhibiting cell proliferation and promoting apoptosis.

Many researchers confirmed that PI3K/Akt/mTOR signaling pathway is related to a few biological activities to affect cell proliferation, survival, and migration [7, 8], especially to stimulate proliferation, survival, invasion/metastasis, and metabolic reprogramming and suppress autophagy, which is involved in possible mechanisms of oncogenic transformation [9]. PI3K/Akt/mTOR signaling pathway is commonly activated and suppression of its activation may inhibit cells proliferation and metastasis in cancers of the lung [10, 11, 50], colorectal [51, 52], esophagus [53], breast [54–56], liver [57, 58], and kidney [59, 60], which has been considered a promising therapeutic target. Whether the PI3K/Akt/mTOR signaling pathway involved in SLBZ-AP affects BMLC was further explored in our study, results of which indicated that the mRNA and protein expression of AKT, mTOR, P70S6, and VEGF as well as protein expression of p-AKT, p-mTOR, p-P70S6, and VEGF was significantly increased in the marrow of tibia from BMLC mice. Rapamycin, also known as Sirolimus, is a specific mTOR inhibitor. Rapamycin could block targets known to be downstream of mTOR such as inhibition of p70S6K activity to inhibit metastatic tumor growth [61, 62]. The phosphorylation of p70S6K has been used as a hallmark of activation by mTOR and correlated with autophagy inhibition in various situations. Pain is one of the major signs of bone metastasis in lung cancer. Furthermore, bone metastasis in lung cancer is associated with tumor cells survival and proliferation. It has been reported in the previous studies that suppression PI3K/Akt/mTOR signaling pathway activation may inhibit cells proliferation and metastasis in lung cancer. In our study, we preliminarily explored the effect of SLBZ-AP on sensitivity to pain in bone metastasis with small-cell lung cancer model mice and cells proliferation and apoptosis of lung cancer as well as possible markers involved. Moreover, signaling by mTOR has been implicated in the development of chronic pain [63]. p-mTOR in the spinal cord may play an important role in the regulation of antinociception induced by acute immobilization stress and the tolerance development induced by chronic immobilization stress [64]. PI3K/p-Akt upregulation may be correlated with chronic osteoarthritis pain [65]. mTOR and its downstream pathway are activated in the dorsal root ganglion and spinal cord after peripheral inflammation which subsequently contribute to the development of chronic inflammatory pain [66]. According to previous studies, rapamycin treatment at the dosage of 4 mg·kg^−1^·day^−1^ was performed in BMLC mice.

Results of our study showed that SLBZ-AP exhibited more antinociceptive and L-D of SLBZ-AP have almost equivalent effects to rapamycin on survival of BMLC mice as well as the enhancement of apoptosis by H-D SLBZ-AP which was similar to that of rapamycin. Similar to rapamycin, SLBZ-AP treatment downregulated AKT, mTOR, P70S6, and VEGF mRNA and protein expression in the marrow of tibia from BMLC mice. Moreover, BMLC-induced protein expression increase of p-AKT, p-mTOR, p-P70S6, and VEGF was downregulated by SLBZ-AP treatment, especially by M-D dosage of SLBZ-AP. However, H-D SLBZ-AP (60 g·kg−1·day−1) tends to show less effect on p-AKT, p-mTOR, p-P70S6, and VEGF protein expression induced by BMLC and the effect of M-D SLBZ-AP is more than that of L-D SLBZ-AP, which may be due to the fact that the expression of p-AKT, p-mTOR, p-P70S6, and VEGF in the marrow from tibia from bone metastasis with small-cell lung cancer (BMLC) mice was measured and cells in the marrow from tibia might include cancer cells and normal cells. The effect of H-D SLBZ-AP remains to be explored further. Rapamycin inhibited BMLC-induced increased expression of mTOR, P70S6, and VEGF particularly, which has equivalent effect on p-AKT, p-mTOR, p-P70S6, and VEGF protein expression to L-D SLBZ-AP although it showed no effect on AKT expression. In our further study, we will perform more behavioral or neurochemical markers of pain and explore the role of chemical components in bone metastasis with small-cell lung cancer.

## 5. Conclusions

In conclusion, with higher incidence rate, BMLC associates with poor prognosis and high morbidity. Furthermore, the patients' quality of life is affected by BMLC-induced pain strongly. PI3K/Akt/mTOR signaling pathway is involved in possible mechanisms of oncogenic transformation as stimulation of proliferation, survival, invasion/metastasis, metabolic reprogramming, and suppression of autophagy and is commonly activated in lung cancer to correlate with cells proliferation and metastasis. As one of Chinese medicine formulas, SLBZ-AP is reported to have curative effect on lung cancer. We investigated the effect of SLBZ-AP on BMLC-induced pain and survival of BMLC mice as well as the involved signaling pathway in present study. Our results indicated that SLBZ-AP relieves BMLC-induced pain and prolongs animals' survival. Results of TUNEL staining indicated that SLBZ-AP treatment promoted cells apoptosis in marrow from tibia of BMLC mice. SLBZ-AP inhibited mRNA and protein expression of AKT, mTOR, P70S6, and VEGF as well as protein expression of p-AKT, p-mTOR, p-P70S6, and VEGF upregulation in the marrow of tibia induced by BMLC, an effect which was similar to rapamycin. Considering that SLBZ-AP showed more effects on pain and survival than rapamycin, and further studies need to be performed. Our results suggested that SLBZ-AP may have antinociceptive effect and prolong survival of BMLC mice at least partially by inhibiting cell proliferation and promoting apoptosis through PI3K/Akt/mTOR signaling pathway. SLBZ-AP may be a potential candidate for BMLC therapy.

## Figures and Tables

**Table 1:** Twenty-eight species of traditional Chinese medicines involved in Shenling Baizhu additive powder (SLBZ-AP).

Chinese name	Latin name^*∗*^	Ratio
Dang shen	Codonopsis Radix	12
Bai Zhu	Atractylodis Macrocephalae Rhizoma	12
Fu Ling	Poria	12
Shan Yao	Dioscoreae Rhizoma	12
Gan Cao	Glycyrrhizae Radix et Rhizoma	6
Bai Bian Dou	Semen Lablab Album	12
Dang Gui	Angelicae sinensis Radix	15
Chuan Xiong	Chuanxiong Rhizoma	15
Bai shao	Paeoniae Radix Alba	15
Sheng Di	Rehmanniae Radix	15
Lian Zi	Nelumbinis semen	10
Yi Yi Ren	Coicis semen	10
Sha Ren	Amomi Fructus	5
Jie Geng	Platycodonis Radix	6
Huang Qi	Astragali Radix	8
Wu Gong	Scolopendra	2
Fu Zi	Aconiti Lateralis Radix Praeparata	10
Rou Gui	Cinnamomi cortex	5
Shan Zhu Yu	Corni Fructus	10
Tu Si Zi	Cuscutae semen	10
Du Zhong Ye	Eucommiae Folium	10
Niu Xi	Achyranthis Bidentatae Radix	10
Lu Jiao Jiao	Cervi Cornus Colla	10
Shi Hu	Dendrobii Caulis	10
Mai Dong	Ophiopogonis Radix	10
Shi chang Pu	Acori Tatarinowii Rhizoma	10
Yuan Zhi	Polygalae Radix	10
Da Huang	Rhei Radix et Rhizoma	6

^*∗*^According to Chinese Pharmacopoeia.

**Table 2:** Primers used in qPCR assay.

Gene	Primer	Primer sequence (5′-3′)	Product
ACTB	Forward	CCCATCTATGAGGGTTACGC	150 bp
	Reverse	TTTAATGTCACGCACGATTTC	

AKT	Forward	CCGAAGGACGGGAGCAG	151 bp
	Reverse	CTCTCAGGCTGGCGCTC	

mTOR	Forward	CTTAGAGGACAGCGGGGAAG	90 bp
	Reverse	TCCAAGCATCTTGCCCTGAG	

P70S6	Forward	ACGAAAGCGCAAGAAATCCC	100 bp
	Reverse	GGAGGCTCCAGGGCATTAGA	

VEGF	Forward	TTCCGAGACAGGGAAGCTGA	273 bp
	Reverse	ATCATTGCCTTTCCATAGCCCC	

## Data Availability

All data generated or analyzed during this study are included in this published article.

## Abbreviations

BMLC: Bone metastasis of lung cancer
SLBZ-AP: Shenling Baizhu additive powder
TCM: Traditional Chinese medicine
EMT: Epithelial mesenchymal transition
SCLC: Human small-cell lung cancer
PI3K: Phosphatidylinositol 3-kinase
mTOR: Mammalian target of rapamycin
IL-2: Interleukin 2
IFN-*γ*: Interferon-*γ*
TNF-*α*: Tumor necrosis factor-*α*.
